# Opportunities for Mobile App–Based Adherence Support for Children With Tuberculosis in South Africa

**DOI:** 10.2196/19154

**Published:** 2020-11-11

**Authors:** Rachel M Morse, Hanlie Myburgh, David Reubi, Ava E Archey, Leletu Busakwe, Anthony J Garcia-Prats, Anneke C Hesseling, Stephanie Jacobs, Sharon Mbaba, Kyla Meyerson, James A Seddon, Marieke M van der Zalm, Dillon T Wademan, Graeme Hoddinott

**Affiliations:** 1 Department of Global Health and Social Medicine King's College London London United Kingdom; 2 Desmond Tutu Tuberculosis Centre Department of Paediatrics and Child Health Faculty of Medicine and Health Sciences, Stellenbosch University Cape Town South Africa; 3 Amsterdam Institute for Social Science Research University of Amsterdam Amsterdam Netherlands; 4 College of Arts and Sciences University of Virginia Charlottesville, VA United States; 5 Social Aspects of Public Health Human Sciences Research Council Cape Town South Africa; 6 Department of Infectious Diseases Imperial College London London United Kingdom

**Keywords:** eHealth, mHealth, tuberculosis, pediatric tuberculosis, adherence

## Abstract

Tuberculosis is the number one infectious cause of death globally. Young children, generally those younger than 5 years, are at the highest risk of progressing from tuberculosis infection to tuberculosis disease and of developing the most severe forms of tuberculosis. Most current tuberculosis drug formulations have poor acceptability among children and require consistent adherence for prolonged periods of time. These challenges complicate children’s adherence to treatment and caregivers’ daily administration of the drugs. Rapid developments in mobile technologies and apps present opportunities for using widely available technology to support national tuberculosis programs and patient treatment adherence. Pilot studies have demonstrated that mobile apps are a feasible and acceptable means of enhancing children’s treatment adherence for other chronic conditions. Despite this, no mobile apps that aim to promote adherence to tuberculosis treatment have been developed for children. In this paper, we draw on our experiences carrying out research in clinical pediatric tuberculosis studies in South Africa. We present hypothetical scenarios of children’s adherence to tuberculosis medication to suggest priorities for behavioral and educational strategies that a mobile app could incorporate to address some of the adherence support gaps faced by children diagnosed with tuberculosis. We argue that a mobile app has the potential to lessen some of the negative experiences that children associate with taking tuberculosis treatment and to facilitate a more positive treatment adherence experience for children and their caregivers.

## Introduction

Tuberculosis (TB) is the number one infectious cause of death globally [1]. Implementation of effective TB control programs continues to place enormous pressure on the health systems of high TB-burden countries [1]. The World Health Organization’s (WHO) Global TB Program identifies digital health technologies as key to supporting its new End TB Strategy [2]. An essential component of this strategy includes using mobile technologies and apps in particular to improve adherence to TB treatment [2]. Reviews show that the development and implementation of mobile health technologies to support health outcomes are overwhelmingly concentrated in high-income countries and focused on diseases of concern in those settings [3-5]. The development and implementation of mobile health technologies in low- and middle-income countries (LMICs), where the burden of communicable diseases, such as TB, is markedly high and treatment is strenuous, has received comparatively less attention. In the last decade, rapid developments in mobile technologies and apps, extended mobile network coverage, and increasing possibilities for integrating mobile health into existing eHealth platforms present opportunities for using widely available technology to support TB programs and adherence in resource-constrained and high-burden areas [2].

In South Africa, TB is the leading cause of death, mainly due to the overlapping HIV and TB epidemics [1,6]. South Africa also has the world’s second highest estimated TB incidence rate, at 520 per 100,000 people [1]. In endemic areas, such as South Africa, young children (especially younger than 5 years) are at the highest risk to progress from TB infection to TB disease and to develop the most severe forms of TB, such as TB meningitis [7,8]. In 2018, children accounted for 7% of newly recorded TB cases in South Africa [1]. Despite their heightened risk, children’s paucibacillary TB disease leads to them having much better treatment outcomes than their adult counterparts once treatment is started [7]. Children’s timely diagnosis and rapid initiation onto TB treatment are critical, as undiagnosed cases or undertreatment could have long-term health consequences or lead to death.

Drug-susceptible (DS) and drug-resistant (DR) TB treatment formulations have had very poor acceptability among children [9]. The drugs are multiple, unpalatable, and often cause significant side effects, such as nausea, liver toxicity, insomnia [1,7], neurosensory alterations, neuromuscular weakness, and psychological and behavioral disturbances such as anger, aggression, withdrawal, hyperactivity, and depressive symptoms [10,11]. TB treatment is also lengthy and requires consistent adherence for at least six months for DS-TB and, in the case of DR-TB, adherence to different drug regimens for a longer period of time [1,8]. Child-friendly TB preventive therapy and treatment formulations that simplify and improve TB treatment for children are either only recently available or are still being evaluated in clinical trials [8,9,12]. The challenges presented above complicate children’s adherence to treatment and caregivers’ daily administration of the drugs [8].

Various small-scale and pilot studies in high-income settings have demonstrated that mobile apps are a feasible and acceptable means of enhancing children’s and young people’s treatment adherence for chronic conditions, such as sickle cell disease (SCD), migraines, asthma, and HIV [13-18]. There is limited high-quality evidence on the impact of mobile apps on health outcomes when implemented at scale or on how different mobile app components affect health and treatment outcomes [3-5]. Despite this, many of the small-scale studies published thus far provide promising evidence supporting the potential of mobile apps to positively impact adherence and treatment outcomes among children and young people [3-5,13,15,16,19]. These studies also serve as a foundation for further research and implementation efforts in this field and demonstrate the potential for developing and implementing mobile apps in LMICs that support children’s and caregivers’ burdensome TB treatment journeys. To date, no mobile apps that aim to improve the experience of TB and TB treatment in high- or low-income countries have been developed for children; mobile health interventions for supporting TB programs have largely focused on adult patients, using SMS-based text message platforms to promote TB treatment adherence, clinic attendance, and monitoring [20-22].

In this paper, we reflect on our joint experience conducting research in ongoing pediatric TB studies at the Desmond Tutu TB Centre, Stellenbosch University, South Africa, to highlight the potential for developing mobile app–based technologies to support TB treatment adherence in children. First, we provide an overview of technologies that have been used to support children’s and young people’s treatment and adherence experiences in other disease contexts. Second, we present hypothetical scenarios informed by our experiences to illustrate the adherence support gaps for children with TB. Third, we imagine how a mobile app could address some of the adherence support gaps faced by children affected by TB.

## Overview of Adherence Technologies for Children

Table 1 provides examples of studies that use mobile technology to improve children’s and young people’s treatment adherence, including for SCD, asthma, migraine, attention-deficit/hyperactivity disorder, and HIV. These pilot studies provide preliminary evidence that shows the potential of mobile apps to support adherence in children and young people. 

**Table 1:** Examples of studies that used mobile technology to improve children’s and young people’s treatment adherence.

Study	Study summary	Intervention features
Creary et al (2019) [13]	- Single-arm intervention study with pediatric patients ≤19 years (median age 10 years) - Condition: SCD^a^ - Country: United States - Intervention: effect of mobile phone–based intervention on adherence to SCD treatment - Outcomes: increased adherence among participants engaged with the intervention over a 6-month period compared with participants’ baseline adherence	- Self-recorded videos of taking the medication - Monetary incentives with successful adherence (US $30 gift card) - Text message reminders - Personalized feedback on adherence from research staff
Ramsey et al (2018) [14]	- AB design pilot study with patients aged 13 to 21 years - Condition: migraine - Country: United States - Intervention: evaluation of the use of a mobile app to increase adherence to migraine medication - Outcomes: similarly high rates of adherence among participants using the intervention and participants using smart pill bottles over an 8-week period. Participants rated the intervention as acceptable and easy to use	- Reminder notifications and phone calls - Incorporation of the health belief model - Medication adherence tracking
Kosse et al (2019) [15]	- Randomized controlled trial with patients aged 12 to 18 years - Condition: asthma - Country: Netherlands - Intervention: measuring the impact of a mobile app on participants’ adherence to asthma treatment - Outcomes: increased 6-month adherence rates among participants who used the app and measured low-adherence rates at baseline. Decreased 6-month adherence rates measured among participants in the control group	- Symptom monitoring - Education (short movie clips) - Medication reminders - Chat feature with pharmacists - Chat feature with peers
Weisman et al (2018) [17]	- Randomized controlled trial with children aged 6 to 16 years - Condition: ADHD^b^ - Country: Israel - Intervention: assessing the use of a mobile app to increase ADHD medication adherence - Outcomes: increased adherence rates among participants who were using the app over 8 weeks compared with adherence rates in the control group	- Symptom monitoring - Reminder notifications - Education on ADHD and its treatment (video clips, articles, and links to additional reading materials)
Curtis et al (2019) [23]	- Study conducted with 12- to 18-year-olds - Condition: SCD - Country: multiple, including Lebanon and Nigeria - Intervention: development of an evidence-based mobile app supporting SCD medication adherence - Outcomes: evaluation of the app is underway	- Avatar - Rewards system (points earned through interaction with app’s features) - Education on SCD - Quiz (focused on education about SCD) - Symptom tracking - Medication and appointment reminders - Emergency information for health professionals unfamiliar with SCD
Hightow-Weidman et al (2018) [18]	- Study conducted with 19- to 24-year-olds - Condition: HIV - Country: United States - Intervention: pilot of a mobile app supporting HIV medication adherence - Outcomes: high acceptability and feasibility, as measured in participant ratings after 28 days of app use. A clinical trial evaluating adherence with the app is underway	- Medication tracking - Reward system (points and monetary incentive earned through interaction with the app’s features) - Social networking (social wall discussion questions) - Gamification through quests and story-based framework - Incorporation of social cognitive theory

^a^SCD: sickle cell disease.

^b^ADHD: attention-deficit/hyperactivity disorder.

However, few technologies and mobile apps target children’s treatment adherence specifically; for TB, no such technologies exist. Common app features shared across the studies included in Table 1 incorporated behavioral and educational strategies to improve adherence through features such as reminder notifications, adherence logging, cash or other adherence rewards, and educational features [13-15,17,18,23]. While evidence of the impact of these app components on adherence is limited to small sample sizes, the studies that have completed evaluations show increased or high rates of adherence in their study populations [13-16,19], and an even greater number of apps have shown high acceptability and feasibility among users from various age categories [13-18]. For example, one of the pilot studies, MyMate&Me, is a mobile app aiming to support SCD medication adherence among children and adolescents aged 12 to 18 years [23]. With MyMate&Me, users earn points by participating in app features, including (1) “Tip of the Day,” where users learn important information about coping with SCD, (2) “Daily Quiz,” where users learn about SCD by answering quiz questions and earning points for correct answers, (3) “Mood Tracker,” where users earn points by logging their daily mood, and (4) “Medication and Appointment Reminders,” where users are reminded to take their medication, attend appointments, and earn points for confirming their adherence and attendance. MyMate&Me also features an avatar to accompany the user throughout the app. The user can spend their earned points on clothes and accessories for their avatar, thereby providing incentive for using the app’s various functionalities and managing their condition. Another study, also on SCD, incorporated video directly observed therapy for adherence monitoring, daily reminders via SMS text messages, email, or telephone if adherence was not observed, and a US $30 gift card incentive if users maintained at least 90% adherence over 30 days [13].

The behavioral strategies used in these mobile apps aimed to facilitate a favorable adherence environment and thus change patient behavior, while the educational strategies aimed to provide patients with information so that they could make informed choices about adhering to treatment [24]. These strategies are informed by underlying theories of human behavior, such as the health belief model [25], the theory of reasoned action or planned behavior [26], and operant conditioning [27]. The basic logic of these theories is that people are more likely to repeat behaviors (in this instance adherence) that (1) are associated with reward, not punishment, (2) conform to their perceptions of descriptive and injunctive norms, and (3) are within their perceived self-control. Robust evidence supporting the use of mobile apps and mobile app features that use behavioral strategies is limited [3-5]; however, their foundational principles have been evaluated extensively in relation to a large variety of health behaviors in many settings across the world [27,28] and have been shown to be supportive of adherence [24]. In 2014, the WHO advocated for such behavioral and educational strategies to be incorporated into apps to improve TB treatment adherence [7].

## What Are the Current Adherence Support Gaps for Children With Tuberculosis?

Many mobile apps that support children’s and young people’s adherence to treatment for other conditions serve as treatment reminders or sources of information for patients [13-15,17,18,23]. Drug palatability, tolerability, and acceptability, which pose serious threats to adherence, are typically not considered for intervention with mobile apps. In Textbox 1, we draw on our experiences carrying out research in pediatric TB studies at the Desmond Tutu TB Centre to consider children’s and caregivers’ experiences with TB treatment that illustrate specific adherence support gaps and needs. The scenarios reflect typical interactions with patients and caregivers that are observed daily in TB service delivery contexts. They show that caregivers and children are generally motivated and knowledgeable about TB treatment but that the unpalatability, number, and side effects of the drugs affect their acceptability and provide serious challenges for caregivers and children at each ingestion event. We argue that an important user need is making the drug ingestion and administration experience more acceptable. For children especially, the current TB treatment experience is negative, and they are “punished” by the negative associations and poor palatability at each ingestion event. Alongside advocating for improved, child-friendly, palatable formulations, a mobile app that can mitigate the punishment associated with treatment and provide tangible rewards closely associated with each adherence event is a priority and could serve as a supporting component for improving the TB treatment experience for children and their caregivers. The app could also, for example, provide users with tutorial videos for pairing TB treatment with foods to improve palatability without compromising dosing accuracy [29].

**Scenarios reflecting specific adherence support gaps and needs.**

**Scenario 1**

Bernice is the mother of 2-year-old Lindiwe and takes her role in administering Lindiwe’s tuberculosis (TB) treatment very seriously. Every day when it is time to administer Lindiwe’s treatment, Bernice endures frightful temper tantrums due to the drugs’ poor taste. Bernice finds that the daily frustration and struggle of ensuring Lindiwe ingests her medication is mentally wearing, and she has had to be increasingly resourceful to plan for and strategize ways of getting Lindiwe to take her treatment, including begging, bribing, forcing, cheering, and more. In this scenario, the adherence gap is Bernice’s struggle to be resourceful and find new ways to administer the TB medication due to the medication’s poor acceptability.

**Scenario 2**

Angela’s 7-year-old son, Henry, often experiences significant nausea when he takes his TB treatment. On occasion, the treatment causes him to vomit. The process of giving Henry his TB medication has become a daily battle for Angela and often involves her forcing the medication into him. This arrangement is beginning to negatively affect their relationship. The adherence support gap in this scenario is the negative effect of the TB medication’s poor palatability and acceptability on Angela’s relationship with Henry.

**Scenario 3**

Despite being only 5 years old, Alice understands that taking TB medication is an important daily task. When it is time for her mother, Gill, to take her own TB medication, Alice reminds her by saying “pills, Mommy, pills.” However, understanding the importance of TB treatment does not help Alice’s adherence; she puts up a great fight each time she has to take her own treatment. In this scenario, the adherence support gap is that Alice’s understanding of the importance of TB treatment is not enough to overcome the poor acceptability of the treatment.

## How Could Technology Address These Gaps?

In order to address the current treatment support gaps for children with TB in South Africa, we conceptualize a mobile app that incorporates behavioral and educational strategies to include an avatar, a rewards scheme, reminder notifications, adherence tracking, social support for children and caregivers, and information on TB, TB treatment, and TB treatment adherence, with the overall aim of reducing the negative emotions associated with TB treatment for children. The connections between treatment support gaps, strategies, and app features are presented in Table 2.

**Table 2:** Summary of adherence gaps and their hypothetical corresponding mobile app features.

Adherence gap	App features: educational	App features: behavioral
Poor acceptability and palatability of TB^a^ treatment for children	Information about the rewards of adherence and the consequences of nonadherence	- An avatar to reduce children’s negative emotions toward treatment and demonstrate the consequences of adherence - Rewards scheme to reduce children’s negative emotions toward treatment - Social support for children from peers
Caregivers’ challenges finding strategies to administer TB treatment	Access to advice and information from health professionals	- Reminder notifications and medication tracking associated with behavior-changing rewards - Social support and advice from other caregivers
Negative effect of the strain of TB treatment on the child-caregiver relationship	Access to advice and information from health professionals	- Rewards scheme to reduce children’s negative emotions toward treatment - Social support and advice for caregivers from other caregivers - Social support for children from peers - An avatar to reduce children’s negative emotions toward treatment
Poor acceptability of TB treatment outweighing the importance of TB treatment for children	Information about the rewards of adherence and the consequences of nonadherence	- An avatar to reduce children’s negative emotions toward treatment and demonstrate the rewards of adherence - Rewards scheme to reduce children’s negative emotions toward treatment - Social support for children from peers

^a^TB: tuberculosis.

We propose that the child creates an avatar to represent themselves within the app. The avatar is given opportunities to thrive (eg, growing noticeably stronger or getting better outfits or gear) by completing tasks so that patients are rewarded for active engagement in their care rather than punished for noncompliance, as is the current norm. The avatar serves as an externalization of the child’s experiences with treatment. If the child cares for their avatar, then the avatar will grow increasingly sophisticated; achieving this is a reward in itself for the child. For example, in such an app, users may complete missions in order to gain diamonds—the currency of the app—that can be used to purchase clothes and accessories for the avatar in the app’s store. Completing missions also leads to levelling up each time the user acquires the required number of diamonds. Each mission might include linked goals, such as following the treatment plan for 3 consecutive days, reading about TB, and joining an online forum discussion. As a user completes missions, earns diamonds, and levels up, their avatar becomes stronger. “Adherence streaks” unlock bonus rewards and items in the app’s store: the longer the adherence streak, the greater the reward. Rewards can be scaled over time so that the user has increasingly higher targets to achieve. As a further incentive, such an app could partner with businesses to offer small tangible rewards, such as mobile data or discounts at selected stores. The app, by encouraging the child’s active participation in their treatment adherence, assigns positive consequences to taking their medication (eg, diamonds, a visibly stronger avatar, and more accessories) and permits the child to gain control and autonomy over the treatment experience. It also ties rewards to adherence despite the unpalatability of the drugs and negative side effects. Interventions that use rewards to promote medication adherence have been shown to be significantly more efficacious than interventions that do not use rewards [30]. The aforementioned features of the app’s design also have the potential to lower the risk of user fatigue. In an app supporting TB treatment adherence, the risk of user fatigue is mitigated, as the need for using the app naturally ends upon treatment completion, at which point the user’s avatar could enter a hall of fame.

Building on a fundamental link between the avatar’s wellness and the child’s adherence behaviors, a mobile app could then incorporate a range of other features to support children and caregivers in adhering to treatment. For instance, the app could be an important source of information on TB. It could include medication reminders, alerts for upcoming clinic visits, and details of what to expect at the visit. Daily adherence logging would allow users to track their adherence over time, view weekly and monthly adherence, and progress toward completing their treatment course. An in-app community space would allow caregivers to interact with other caregivers. Caregiver participation in the community would earn their child diamonds, making knowledge seeking collaborative and providing caregivers with a new adherence resource and a free, positive reward to help mitigate the negative effects of forced administration on the child-caregiver relationship. In the online forum, caregivers and older children with appropriate supervision would be able to ask each other about their experiences with TB and treatment, creating an additional social support system.

Studies of TB knowledge in South Africa have suggested relatively good levels of understanding of prevention and cure among the population, but knowledge of TB cause and symptoms was suggested to be low [31,32]. A frequently asked questions (FAQs) section, designed by children and adolescents who have experienced TB disease and medical professionals who work with TB disease, could address knowledge gaps by including answers to common questions, such as “How can I protect my family and friends from getting TB?” “What kind of bodily changes can I expect while taking treatment?” “Can I go to school if I have TB?” and “What is the relationship between TB and HIV?” In the app’s community space, users can also play games (racing, sports, treasure hunts, etc), either with each other or solo, to earn additional diamonds. The stronger their avatar (eg, the better their adherence), the more competitively they will perform in the games. The effectiveness of such games for increasing medication adherence among children has been shown [33,34]. For example, in a randomized controlled trial, a video game intervention was found to improve medication adherence and behavioral outcomes for young people aged 13 to 29 years with acute leukemia, lymphoma, or soft-tissue sarcoma in the United States, Canada, and Australia [33].

In a high TB-burden, resource-limited setting like South Africa, such a mobile app that aims to facilitate children’s adherence to TB treatment could improve the patient experience and also potentially buffer some of the shortcomings of the care provided in an overburdened health service. In particular, a mobile app could offer avenues of support to caregivers and children that are not immediately available in service encounters. It could also be a source of information on a range of topics related to the disease and its treatment, as in the FAQ section proposed. The immediate availability of such support and information has the potential to be empowering for patients.

## Considerations for Implementation

There are a number of practical considerations for developing and using mobile apps to support children’s adherence in culturally diverse, developing health settings, such as South Africa. First, most households in low socioeconomic settings have limited access to phones and tablets that could support apps. Programs may consider providing patients with smartphones during treatment, with the possibility of taking ownership when treatment has been completed successfully. The resources required to deliver such an app (if effective in improving adherence) are limited to the supply of a suitable mobile device (about US $40 per user) and some training on how to use the app. Studies suggest that the cost of providing smartphones to patients and supporting app-based adherence is more cost-effective for patients and health services than current adherence support models, which are often highly labor-intensive, requiring many hours of health workers’ time [35,36]. The app would also require some tailoring to the age of the child to provide age-appropriate services and support, including features for older children and adolescents who may experience greater treatment autonomy. During the app’s initial setup, a prompt to input age can align the app’s functionality with the age of the child. To be acceptable amongst diverse users, the app must be attuned to the local context in which it will be implemented. As such, the app’s avatar must offer an array of characteristics that users can select to reflect their social, cultural, racial, and gender identities and their personalities. These could be informed by participatory research through focus group discussions and key informant interviews with end users, community advisory boards, patient advocacy groups, and other stakeholders. The project team can then partner with an expert in app design, development, and programming. Last, the app should take into account the diversity of languages spoken in the local context and thereby allow children and caregivers to use the app in the language most suitable to them.

Given the paucity of robust evidence for using mobile apps to support adherence in children and adults, we suggest that future evaluations of mobile adherence apps to support children’s adherence to TB treatment should include implementation science evaluations and randomized controlled trials. Further, such evaluations should measure both biomedical (eg, treatment success) and patient experience (eg, preferences and user satisfaction) outcomes.

## Conclusion

Digital health technology is a burgeoning resource for supporting health systems and patients in the prevention, treatment, and care of a wide array of diseases. While such technologies hold promise for supporting the adherence of children diagnosed with TB disease in LMICs, where the burden is highest, no such technologies have been developed to date. In this paper, we presented some hypothetical scenarios of the daily struggles of caregivers and children who are tasked with adhering to TB treatment for prolonged periods of time. While we do not suggest that a mobile app will resolve all problems related to children’s adherence to treatment, such a technology could compensate for and lessen some of the negative experiences children associate with taking TB treatment and perhaps even act as a buffer for health system shortcomings. It remains the task of the research community to develop such technologies and bring them to scale in the health systems in which they will have the greatest impact.

## Abbreviations

DR: drug-resistant
DS: drug-susceptible
FAQ: frequently asked question
LMIC: low- and middle-income country
SCD: sickle cell disease
TB: tuberculosis
WHO: World Health Organization
